# Acetaminophen (Paracetamol): Use beyond Pain Management and Dose Variability

**DOI:** 10.3389/fphys.2017.01092

**Published:** 2017-12-22

**Authors:** Christopher J. Esh, Alexis R. Mauger, Roger A. Palfreeman, Haifa Al-Janubi, Lee Taylor

**Affiliations:** ^1^Aspetar – Qatar Orthopaedic and Sports Medicine Hospital, Athlete Health and Performance Research Centre, Doha, Qatar; ^2^Endurance Research Group, School of Sport and Exercise Sciences, University of Kent, Chatham Maritime, Chatham, United Kingdom; ^3^Aspetar – Qatar Orthopaedic and Sports Medicine Hospital, Exercise and Sports Science Department, Doha, Qatar; ^4^Aspetar – Qatar Orthopaedic and Sports Medicine Hospital, Pharmacy Department, Doha, Qatar; ^5^School of Sport, Exercise and Health Sciences. Loughborough University, Loughborough, United Kingdom

**Keywords:** acetaminophen, cyclooxygenase, prostaglandin E2, antipyretic, analgesic

## Introduction

Inappropriate prescribing of analgesics or self-administration through over the counter formulations results in regular often negative reporting within the media. Indeed, their abuse within elite through to recreational sport is not uncommon (Feucht and Patel, 2010; Warden, 2010; Tscholl and Dvorak, 2012; Brewer et al., 2014). For example, acetaminophen (ACT; commonly known as paracetamol) use is even prevalent in young sub-elite athletes, who consume ACT or other analgesics [e.g., non-steroidal anti-inflammatory drugs (NSAIDs)] to decrease pain from previous athletic exertion or prophylactically to reduce pain in subsequent training/competition (Garcin et al., 2005). Related recent headlines include a former FIFA Chief Medical Officer stating that “about half of players competing at the past three World Cups routinely took NSAIDs like ibuprofen” whilst former players report pressure to use such drugs “to get through games” (BBCSport, 2017). NSAIDs and ACT are the most commonly used antipyretic and analgesic drugs worldwide (Hinz and Brune, 2012). Given they are available over the counter, their use even with careful club/team physician oversight can be difficult to police within a professional sport environment. If team clinicians ban over the counter analgesia use, players can and do simply self-administer, increasing risk of drug interaction with clinician prescribed medications and/or over-dose risk (inadvertently or otherwise).

ACT has been used globally since 1955 and is considered a safe effective analgesic (Bunchorntavakul and Reddy, 2013). The analgesic properties of ACT are similar to NSAIDs, yet ACT does not share similar NSAID mediated anti-inflammatory actions (Graham et al., 2013). ACT carries fewer, if any, side-effects when used at appropriate dosages compared to NSAIDs (Towheed et al., 2006; Jones et al., 2015). NSAIDs side-effects can include gastrointestinal and arterial wall damage, gastrointestinal ulcers, arterial blood clotting, myocardial infarction, stroke and hemorrhage perforation, amongst others (Ong et al., 2007; Van Wijck et al., 2012). The well evidenced and publicized side-effect of ACT is liver necrosis in cases of acute overdose [e.g., ACT at 250 mg.kg, ~15 g for a 60 kg human (Prescott et al., 1971; Prescott, 1980; Raffa et al., 2014)], evidently not seen under appropriate acute and/or regular use (Skoglund et al., 1991; Dippel et al., 2003a). Although uncommon relative to ACT chronic use, liver necrosis has been reported when concomitant with other factors such as fasting/malnutrition, alcoholism and the use of cytochrome P450 enzyme inducing drugs (Vitols, 2003).

Novel research has seen ACT used outside of its typical clinical parameters, including exercise performance domains (Mauger et al., 2010, 2014; Foster et al., 2014) and to induce mild hypothermia within afebrile humans (Foster et al., 2016, 2017). ACT use whilst safe in many scenarios (Ong et al., 2007; Graham et al., 2013) does present risk in others (Hinz and Brune, 2012; Aminoshariae and Khan, 2015), across research and clinical agendas. This opinion piece will detail the variable acute dose and chronic dosages, and their pharmacological safety across established and emerging research fields. In particular, this article will present evidence to support the following opinions:

1. ACT should not be advocated for use within a sporting context, given the present evidence is from tightly controlled laboratory studies which do not replicate the multifaceted physiological, biochemical, and environmental perturbations to homeostasis during sporting performance.
2. ACT may provide a useful tool in determining fundamental biochemical thermoregulatory mechanisms *in vivo* in humans, when appropriate experimental controls and designs are employed.

## Emerging acetaminophen use within physiology research

Evidence presented/discussed herein focuses on orally administered ACT in adult populations (unless stated otherwise). An “acute” ACT dose constitutes one-time single administration, whilst a chronic ACT “dosage” describes multiple doses over a day(s). An adult therapeutic dose of ACT is a 1 g single dose or a dosage of 4 ^*^ 1 g daily at 4–6 h intervals (Bunchorntavakul and Reddy, 2013). Children aged 2–12 years are normally given an individualized body weight derived therapeutic dose at 10–15 mg.kg (Jackson et al., 1984). ACT has been used beyond its primary functions as an analgesic and antipyretic in attempts to understand or augment athletic performance (Mauger et al., 2010, 2014; Mauger and Hopker, 2012; Foster et al., 2014) and manipulate/characterize thermoregulation within temperate, cold and hot environments (Dippel et al., 2001, 2003b; Coombs et al., 2015; Taylor et al., 2015; Foster et al., 2016, 2017). From these specific fields of research, no evidence of adverse reactions to ACT have been reported. The evidence informed safety of ACT from seminal work across a variety of populations will be discussed later in this article (see ACT dose and pharmacokinetics section).

An acute ≤ 1.5 g ACT dose has been used in athletic performance research to improve time trial, time to exhaustion and repeated sprint exercise performance (Mauger et al., 2010, 2014; Mauger and Hopker, 2012; Foster et al., 2014). These effects were attributed to ACT-mediated reductions in exercise induced pain, resulting in maintenance of a higher power output and/or an increased time to exhaustion, despite no change in perceived pain or exertion compared to a placebo condition (Mauger et al., 2010, 2014; Foster et al., 2014). Whilst tolerance of exercise induced pain can be crucial to athletic performance (Ayoub et al., 2004; Amann et al., 2009; Astokorki and Mauger, 2017a,b), it also serves to provide essential feedback on injury and/or fatigue [i.e., a deterrent/modulator of an activity potentially causative of harm (Mauger, 2013)]. Indeed, complete inhibition of pain elicited adoption of an over-aggressive sub-optimal cycling pacing strategy resulting in greater peripheral fatigue and no improvement in athletic performance, without injury pesentation (Amann et al., 2009). This suggests that pain may provide valuable feedback to higher centers to maintain an “optimal” distribution of work rate (Ayoub et al., 2004; Astokorki and Mauger, 2017a,b; St Clair Gibson et al., 2017).

Disruption of thermoregulatory mechanisms by ACT have been linked to improved time to exhaustion during cycling exercise in the heat (Mauger et al., 2014). Attributed to core temperature (Tc), skin temperature, body temperature and thermal sensation being significantly reduced in the ACT compared to placebo condition (Mauger et al., 2014). This suggests ACT disrupted thermoregulatory mechanisms and reduced thermal strain during exercise (Mauger et al., 2014), as observed elsewhere (Burtscher et al., 2013; Veltmeijer et al., 2016). These specific thermoregulatory effects of ACT may, or may not, have been complemented by the analgesic effects discussed above (Mauger et al., 2010; Foster et al., 2014). It must be stated here that the above exercise performance discussions are not advocating the use of ACT within these domains. The clinician and athlete (elite or otherwise) together should deliberate the ethical and safety considerations of strategies that override homeostatic controls before implementing them to enhance exercise performance, or indeed for any other purpose (clinically or otherwise).

ACT can alter thermoregulation in clinical (Dippel et al., 2001, 2003b; Koennecke and Leistner, 2001; Kasner et al., 2002) and healthy populations (Foster et al., 2016, 2017). Up to half of patients who suffer an acute stroke present with a febrile Tc (Dippel et al., 2001). ACT administration elicited dose dependent Tc reductions of 0.4°C [6 ^*^ 1 g/day for 5 days (Dippel et al., 2001, 2003b)] and 0.22°C [3.9 g/day for 5 days (Kasner et al., 2002)] in these patients. ACT ingestion [20 mg.kg lean body mass (LBM)] induced mild hypothermia (range: 0.1–0.39°C drop in Tc) in afebrile participants housed in ambient conditions of 20°C 40% relative humidity [RH (Foster et al., 2016)]. Such conditions are below the human thermoneutral zone (TNZ) where the body invokes mechanisms to maintain a stable Tc (Stocks et al., 2004). Within a similar design to Foster et al. (2016), Foster et al. (2017) demonstrated within cold (10°C 40% RH) compared to thermoneutral conditions (25°C 40% RH), ACT ingestion (20 mg.kg LBM) elicited Tc instability (Foster et al., 2017). Specifically, Tc decreased in the cold condition (10°C 40% RH) with ACT ingestion (range: 0.16–0.57°C) and remained stable within the cold placebo condition whilst Tc stability was maintained in both placebo and ACT thermoneutral conditions (25°C 40% RH). This *in vivo* human data suggest ACT has inhibitory actions on heat producing mechanisms in certain febrile (Dippel et al., 2001, 2003b; Koennecke and Leistner, 2001; Kasner et al., 2002) and afebrile states (Foster et al., 2016, 2017). Within afebrile states the limited data suggests hypothermic effects present only below the human TNZ (Foster et al., 2016, 2017).

Mechanistically it appears ACT has an ability to disrupt “normal” thermoregulatory mechanisms in humans beneath the TNZ (Foster et al., 2016, 2017). This is outside ACT's typical use as an analgesic and febrile antipyretic. Indeed, whilst explicitly described within rodents (Langenbach et al., 1995; Ayoub et al., 2004), the cyclooxygenase (COX)-prostaglandin E2 (PGE2) thermogenesis pathway (detailed below) has not been robustly determined *in vivo* within humans. COX1 knockout mice produced 99% less PGE2 than their wild type counterparts (Langenbach et al., 1995). No compensatory COX2 production to maintain PGE2 levels occurred when inflammation was not present (Langenbach et al., 1995). Such data indicate that PGE2 production, and thus thermogenesis, is somewhat dependent upon COX1 in the absence of a febrile and/or inflammatory state. Confirming the thermogenic influence of this COX1-PGE2 pathway in afebrile and non-inflammatory mammal's, housed beneath their TNZ, is a robust dose dependent drop in Tc within wild type mice [i.e., not COX1 knockout mice (Ayoub et al., 2004; Fukushima et al., 2017)]. ACT administration in the largest doses (300 mg.kg) produced the greatest Tc decreases [2 and 4°C (Ayoub et al., 2004; Fukushima et al., 2017)], and a 96% reduction in PGE2 concentrations (Ayoub et al., 2004). There is uncertainty within the literature on the function of a third COX isoform within mammals, a splice variant of COX1, COX3. This data suggests that COX3 could be the primary target of ACT (Botting, 2000; Chandrasekharan et al., 2002; Botting and Ayoub, 2005). These COX1-3 (Chandrasekharan et al., 2002; Botting and Ayoub, 2005) and/or PGE2 (Langenbach et al., 1995; Ayoub et al., 2004) results were generalized to humans without specific evidence (Kis et al., 2005) given the analgesic and antipyretic functions of ACT are closely related to COX2 inhibition (Graham and Scott, 2005; Graham et al., 2013). The human specific general consensus within the literature, is that the inhibition of COX isoforms, COX1 (maintains homeostasis) and COX2 [combats pathophysiological states including inflammation, pain and fever (Fitzpatrick, 2004; Hinz et al., 2008; Rouzer and Marnett, 2009; Hinz and Brune, 2012; Graham et al., 2013; Józwiak-Bebenista and Nowak, 2013)] alter “normal” thermoregulatory physiological responses/controls. Yet this consensus is derived from rodents, albeit robustly (Langenbach et al., 1995; Ayoub et al., 2004), and not substantiated *in vivo* in humans. Research to confirm such consensus in humans is paramount given the wide variance of responses to ACT observed between and even within species (different strains) of laboratory animals (Bessems and Vermeulen, 2001). In light of the evidence from Foster et al. (2016), Foster et al. (2017) and related hypotheses (Foster et al., 2015) future well-controlled experimental trials, *in vivo* in humans, utilizing ACT to inhibit COX could characterize COX1-3 and PGE2 concentrations and their mechanistic relationship with thermogenesis.

Given the increasing prevalence of research conducted using ACT outside of its typical functions (Mauger et al., 2010, 2014; Mauger and Hopker, 2012; Foster et al., 2014, 2016, 2017; Coombs et al., 2015) the matter of pharmacological safety is current, relevant and important. A plethora of evidence is available highlighting the safety of ACT, including doses/dosages beyond (e.g., 20 mg.kg, 20 mg.kg LBM, up to 2 g) the typical therapeutic range described earlier. Despite this, ACT often receives seemingly unwarranted negative exposure. This is likely due to a high incident rate of acute liver failure in cases of overdose, which accounts for 50% of all acute liver failure cases in the United States (Bunchorntavakul and Reddy, 2013; Yoon et al., 2016). In these ACT acute liver failure cases, treatment with ACT antidote N-acetyl cysteine produces low estimated mortality rates of 0.4% (Bunchorntavakul and Reddy, 2013; Yoon et al., 2016). Without rapid treatment of an overdose the risk of acute liver failure and death rises significantly (Blieden et al., 2014). The minimum acute ACT dose observed to induce acute liver failure is 125 mg.kg body mass (Prescott, 1983). However, incidents are more commonly observed when doses exceed 250 mg.kg body mass [equivalent to 15 g for a 60 kg human (Prescott, 1983)], far greater than that utilized in recent novel applied research [20 mg.kg LBM up to 1.5 g (Mauger et al., 2010, 2014; Mauger and Hopker, 2012; Foster et al., 2014, 2016, 2017; Coombs et al., 2015; Taylor et al., 2015)]. From chronic use of ACT however, there are several incidents of hepatotoxicity from chronic therapeutic doses but these cases are concomitant with other drug use, periods of starvation, other illnesses and apparent allergic reactions (Vitols, 2003). When proposed for use within a research context and the dose exceeds what is typically considered therapeutic (1 g), ACT is often treated with non-evidence-informed scrutiny by ethical review boards. The next section of this article seeks to summarize existing literature and provide evidence of safe ACT doses/dosages, their pharmacokinetics and highlight the minimal risk ACT poses to health within research, when evidence informed dose/dosages are utilized.

## Acetaminophen dose and pharmacokinetics

High rates of overdose incidents are likely due to the ease of access to ACT over the counter, (Bunchorntavakul and Reddy, 2013; Yoon et al., 2016). Taken orally ACT is rapidly metabolized in the gastrointestinal tract and then primarily by the liver into toxic and non-toxic compounds (Hodgman and Garrard, 2012). Absorption through the gastric mucosa is negligible but rapid from the small intestine and thus the rate of absorption is determined by the rate of gastric emptying (Prescott, 1980). Whilst gastric emptying can be slowed by food intake the total amount of ACT absorbed over time would not be affected (Prescott, 1980). The volume of distribution once in general circulation, in healthy adults, is circa 50 l (Forrest et al., 1982). Seminal and emerging research provides substantial evidence that ACT, even administered above the therapeutic dose (1 g), is safe. Within healthy, and some clinical populations, doses/dosages up to 2 g acutely and 2 g 4 ^*^ daily over multiple days have been used within research (Forrest et al., 1979; Benson, 1983; Skoglund and Pettersen, 1991; Skoglund et al., 1991; Dippel et al., 2001, 2003b). The plasma half-life of acute therapeutic (1 g) and larger doses of ACT (20 mg.kg up to 1.5 g) ranges between 1.5 and 4 h in humans, with 95% of the dose excreted via urination 24 h post ingestion as unchanged ACT or conjugated with glucuronic acid, sulphuric acid, mercapturic acid and cysteine (Prescott et al., 1971; Forrest et al., 1979, 1982; Prescott, 1980). Only in those with clinical liver damage is ACT plasma half-life prolonged up to and in excess of 4 h (Prescott et al., 1971; Forrest et al., 1979). Typically within clinical trials the zenith in plasma concentrations reach 10–25 μg/ml 1–2 h post ingestion (Slattery et al., 1987; Singla et al., 2012). A well-controlled pharmacokinetic study in healthy individuals saw peak plasma concentrations of 8–19 μg/ml from an individualized dose (20 mg.kg LBM) up to ~1.5 g (range: 1.019–1.42 g), reached within 120 min of oral ingestion (Foster et al., 2016). If peak plasma concentrations exceed 300 μg/ml liver damage is likely (Prescott, 1983). If plasma concentrations of 200 μg/ml 4 h post ingestion are seen the risk of developing hepatotoxicity significantly increases (Bunchorntavakul and Reddy, 2013). To contextualize, acute therapeutic doses (1 g) or those slightly larger (20 mg.kg up to 1.5 g) produce plasma concentrations of 8–25 μg/ml (Rawlins et al., 1977; Slattery et al., 1987; Singer et al., 1995; Foster et al., 2016). This is some 175 μg/ml lower than that typically associated with a risk of hepatotoxicity (Bunchorntavakul and Reddy, 2013) and 275 μg/ml lower than the established thresholds for liver damage (Prescott, 1983). Liver damage in response to therapeutic ACT doses have only been seen in very specific populations—those who are alcohol dependent, patients receiving enzyme-inducing drugs and those with specific infectious diseases such as measles and infectious mononucleosis (Collins and Starmer, 1995; Zimmerman and Maddrey, 1995; Prescott, 2000; Aminoshariae and Khan, 2015). Indeed, doses in the range of 1–1.5 g (when a 20 mg.kg LBM dose is employed) in exercise performance research (Mauger et al., 2010, 2014; Mauger and Hopker, 2012; Foster et al., 2014) and 20 mg.kg LBM (up to a maximum of 1.5 g) in thermophysiology literature (Foster et al., 2016, 2017) have been used, without reported side-effects; not unexpected given the previous comments regarding plasma ACT concentrations and liver damage. Indeed, 2 g acute doses (even when repeated within-day) have been tolerated without liver-related or any other side-effects (Skoglund and Pettersen, 1991). Despite this, ACT receives negative reactions when proposed for use within a research context regarding acute doses outside a range that is typically considered therapeutic (1 g). Even with evidence suggesting these are entirely safe (e.g., 20 mg.kg, 20 mg.kg LBM, or 1.5 g) provided they are not administered to the clinical populations outlined above and the fact that such doses have already been utilized regularly elsewhere (Skoglund et al., 1991; Dippel et al., 2001, 2003b; Mauger et al., 2010, 2014; Foster et al., 2014, 2016, 2017).

Whilst no side-effects were reported from acute 2 g (Skoglund and Pettersen, 1991) and 20 mg.kg LBM (up to 1.5 g maximum) ACT administration (Foster et al., 2014, 2016; Mauger et al., 2014; Coombs et al., 2015), gastrointestinal damage or liver necrosis were not quantitatively assessed. However, Benson (1983), Dippel et al. (2001), Dippel et al. (2003b) and Forrest et al. (1979) who implemented a range of liver function tests (aspartate aminotransferase, alanine aminotransferase, alkaline phosphatase and total bilirubin levels) demonstrated no liver-related side-effects using similar doses. Chronic dosages exceeding the therapeutic dose (6 ^*^ 1 g/day) over five days in acute ischemic stroke patients (Dippel et al., 2001, 2003b) saw six adverse liver events in the ACT and ibuprofen condition and three in the placebo condition (Dippel et al., 2003b). Overall adverse events (gastrointestinal damage, pneumonia, progressive stroke) were seen equally across ACT, ibuprofen and placebo condition (Dippel et al., 2001, 2003b) and therefore could not be fully attributed to the treatment with ACT. Furthermore, it must be emphasized that this data (Dippel et al., 2001, 2003b) is from a population presenting with pathophysiology (i.e., acute ischemic stroke) that increases the biomarkers typically associated with the diagnosis of liver related “adverse events.” For example, an increase in direct bilirubin is correlated with the severity of stroke (Pineda et al., 2008; Luo et al., 2012; Muscari et al., 2014). Indeed, even within patients with stable, mild and severe chronic liver disease, ACT administration (1.5 g acute, 4 ^*^ 1 g/day for 5 and 14 days) has not been shown to exacerbate liver damage (Forrest et al., 1979; Benson, 1983). Rather prolongation of the ACT half-life is seen (Forrest et al., 1979; Benson, 1983), which if not accounted for could lead to accumulative plasma concentrations of ACT beyond thresholds typically considered “safe” [(e.g., 200 μg/ml (Bunchorntavakul and Reddy, 2013)] resulting in inadvertent overdose and thus further liver damage.

From the evidence presented here the standard therapeutic dose of ACT (1 g single dose up to 4 g daily every 4–6 h) has not been reported to induce liver damage (Benson, 1983). Acute doses up to 1.5 g and dosages up to 6 g/day have been shown to be safe within clinical (Forrest et al., 1979; Dippel et al., 2001, 2003b) and healthy athletic populations (Foster et al., 2014, 2016, 2017). Evidently with such an excellent safety record these dose/dosages should not be considered a significant risk in research studies. However, various control/safety measures have been employed or could be recommended to provide further reassurance of these albeit small risks for research participants. These could include: (1) utilization of alcohol use disorder identification test (AUDIT; as part of participant inclusion criteria for enrolment onto a research project); (2) breathalyzer use (prior to ACT ingestion); (3) the satisfactory completion of an ACT risk assessment questionnaire (Foster et al., 2016, 2017). Following these processes if the researcher or participant are unsure of their suitability then medical clearance can be sought. These recommendations could act to reduce the already minimal risk of ACT-mediated side-effects in apparently healthy populations. Their adoption within germane ACT centric research designs should appease any ethically orientated and/or participant safety concerns. Indeed, such measures have seen use elsewhere already (Foster et al., 2016, 2017).

## Summary and conclusions

From the evidence presented here ACT does not pose a major threat to health, particularly compared to the adverse events seen with NSAID use, unless taken in inappropriate dose/dosages and/or by certain clinical populations. Experimental controls have been suggested to reduce the already low side-effect risk from ACT administration within relevant human experimental designs. While there is evidence of ACT being beneficial during exercise, this is within a well-controlled research setting where interactions with other medication and supplements (NSAIDs, caffeine, etc.), other common conditions (redistribution of blood flow, hydration status, diet, etc.) and the intensity of exercise are explicitly controlled to ensure the safety of participants. ACT use during exercise where such robust controls are not in place must not be advocated, the risk of interaction with any of the stated factors is not known and may increase the danger of injury/damage to an individual. ACT may be a useful tool to explore and validate bio-chemical thermophysiological mechanisms within afebrile human populations. ACT may alter heat producing mechanisms via inhibition of COX and subsequently PGE2, which requires evidence *in vivo* from humans in lieu of rodent evidence discussed above. Drugs that have COX inhibiting mechanisms, are associated with potentially severe adverse side-effects (Ong et al., 2007) although, ACT has been shown to have a favorable risk profile compared to other common COX inhibiting drugs such as NSAIDs, particularly relative to gut damage (Towheed et al., 2006; Jones et al., 2015). Given the propensity of exercise stress to induce endotoxemia, intestinal hypoperfusion, and intestinal cellular injury/damage (van Wijck et al., 2011; ter Steege et al., 2012; March et al., 2017), a result of redistribution of blood flow during exercise compromising the intestinal mucosa wall (Van Wijck et al., 2012; Yeh et al., 2013), such damage could be incurred if ACT was to be used during uncontrolled exercise (i.e., outside of research) within any environment (although most likely a hot environment). However, no reports of any adverse effects have occurred thus far in the literature (Mauger et al., 2014; Coombs et al., 2015). Therefore, the potential for ACT to cause any intestinal injury must be established within experimental designs that explore ACT mechanisms during exercise and/or challenging thermal environments. The evidence discussed above rationalizes the following two opinions:

1. ACT should not be advocated for use within a sporting context, given the present evidence is from tightly controlled laboratory studies which do not replicate the multifaceted physiological, biochemical and environmental perturbations to homeostasis during sporting performance.
2. ACT may provide a useful tool in determining fundamental biochemical thermoregulatory mechanisms *in vivo* in humans, when appropriate experimental controls and designs are employed.

## Author contributions

All authors listed have made a substantial, direct and intellectual contribution to the work, and approved it for publication.

### Conflict of interest statement

The authors declare that the research was conducted in the absence of any commercial or financial relationships that could be construed as a potential conflict of interest.
